# Transcription factor c-Rel regulated by E5 affects the whole process after HPV16 infection through miR-133a-modulated feedback loop aim at mir-379-369 cluster

**DOI:** 10.1186/s12935-022-02794-6

**Published:** 2022-12-01

**Authors:** Juan Zhou, Yongpeng Li, Ke Xu, Yan Rong, Siting Huang, Hailun Wu, Xianlin Yi, Chanzhen Liu

**Affiliations:** 1 Department of Gynecologic Oncology, Guangxi Medical University Cancer Hospital, Nanning, 530021 Guangxi China; 2 Department of Urology, Wuming Hospital of Guangxi Medical University, Nanning, 530199 GuangXi China; 3 Department of Urology, Guangxi Medical University Cancer Hospital, Nanning, 530021 Guangxi China; 4 Department of Gynaecology and Obstetrics, Wuhan Children’s Hospital (Wuhan Maternal and Child Healthcare Hospital), Tongji Medical College, Huazhong University of Science and Technology, Wuhan, 430016, Hubei, China

**Keywords:** Transcription factor, Cervical lesion, Human papilloma

## Abstract

**Background:**

During the development of cervical cancer, HPV infection causes a series of changes in transcription factors and microRNAs. But their relationships with pathogenic processes are not clear.

**Methods:**

Base on previous study, to analyse the relationship among HPV16 infection and the related transcription factors, related miRNAs, so as to further understand the molecular mechanism of HPV16 infection to cervical cancer, around the HPV16 related miRNAs we have reported, the methods of bioinformatics prediction, histology, cell model in vitro and molecular interaction were used for prediction and validation respectively

**Results:**

The results showed that NF-κB family members(c-Rel, p65 and p50) were identified as main HPV16rmiR-transcription factors. They have different expressive characteristics in cervical lesions and play tumorigenesis or progression roles in different periods of HPV16 infection. c-Rel, p65 and p50 act as mediators which link the HPV16 E5 and HPV16 related miRNAs. Among them, c-Rel affects the occurrence and progression of cervical cancer during whole HPV16 infection stage through miR133a-3p–modulated mir-379-369 cluster with a positive feedback way which targeted c-Rel itself and its positive regulator AKT3.

**Conclusion:**

So in the course of HPV16 infection, the E5, c-Rel, and miR-133a-3p form a positive feedback system which aim at mir-379-369 cluster for the whole process from HPV16 infection to cervical cancer.

**Supplementary Information:**

The online version contains supplementary material available at 10.1186/s12935-022-02794-6.

## Background

Cervical cancer is one of the most common malignancies in women globally which threatens women’s health seriously. As the most important pathogenic agent, high-risk human papillomavirus(hr-HPVs) is responsible for the initiation and progression of more than 95% cervical cancer while HPV16 infection is associated with 50% cervical cancer approximately [1, 2]. However, the occurrence and development of HPV-induced cervical cancer is a gradual process of cumulative effect due to the continuous imbalance of host cell oncogenes and tumor suppressor genes accompanied by the abnormal changes of many important cell signaling pathways, which may occur much earlier than the cervical morphological changes [3], and lead to about 10% of persistent hr-HPV infections induce cervical epithelial canceration and eventually transform into clinical cervical cancer [4].

In the previous study, we first proposed that there are two groups of microRNAs(miRNA) act as tumor suppressors which down-regulated in HPV16 positive cervical carcinoma (termed as HPV16 related microRNA, HPV16rmiR): the first group demonstrates a stepwise down-regulation from normal cervix samples to HPV16-infected cervix samples, whereas the other group is exclusively down-regulated when the cervix is transformed to a cancerous state [5]. This finding suggests that the first group of miRNAs may represent the early targets of HPV16 infection when the cervical tissues still appear normal and are not malignant. The aberrant expression of this group of miRNAs might gradually transform the normal cervix to a precancerous state. When the infected cervix becomes cancerous, the expression of the second group of miRNAs change, accompanying or causing abnormal cellular process. That is to say, these two groups of miRNAs are downregulated in early and late phase of HPV16 infection respectively, the aberrant expression of the first group of miRNAs might gradually induce the second group of miRNAs change and then transform the normal cervix to a cancerous state, but that has not been confirmed.

As important regulators of miRNA [6], transcription factors(TF) promote or inhibit gene expression at the transcriptional level by identifying and binding to specific sequences of target genes’ promoter regions, are key links in various cellular signaling pathways through gene overexpression or silence [7]. Prediction via bioinformatic databases showed that NF-κB family members are the most probable upstream transcription factors of these miRNAs mentioned above (termed as HPV16 related microRNA upstream transcription factor, HPV16rmiR-TF), may play vital mediating roles at different stages in the series process of HPV16 infection, relevant miRNAs down-regulation, and subsequent progression of cervical lesions formation and development. Similar to other types of cancer [8, 9], there are TFs-miRNAs regulatory networks in the process of cervical cancer.

So in the present study, around these HPV16 related miRNAs, we first predicted the TFs-miRNAs-target genes network through biological information methods. After verification of the predicted results, NF-κB family members(c-Rel, p65 and p50) were identified as main HPV16rmiR-TFs. By histological staining, they show different expressive characteristics in cervical lesions and play tumorigenesis or progression roles in different periods of HPV16 infection. Through molecular interaction study, c-Rel, p65 and p50 were found that act as mediators which link the HPV16 and two groups of miRNAs. Among them, c-Rel affects the occurrence and progression of cervical cancer during whole HPV16 infection stage through miR133a-3p–modulated mir-379-369 cluster with a positive feedback way through c-Rel itself and miR329-5p target gene AKT3. All the results exhibited new interpretation of the pathogenesis and progression of cervical cancer.

## Methods

### Cervical tissues

The informed consent of relevant research subjects and ethical review consent from the ethics Committee of Guangxi Medical University Cancer Hospital have been obtained before research. A total of 215 cases of paraffin tissue samples from patients were collected in gynecology department from November 2011 to May 2014, including 116 cases of cervical cancer tissues derived from radical bilateral hysterectomy ± pelvic lymph node dissection ± abdominal aortic lymph dissection; 22 cases of low grade squamous intraepithelial lesions (LSIL) and 26 cases of high grade squamous intraepithelial lesions (HSIL) were obtained from conization of uterine cervix of precancerous lesion at the same time; 51 cases of cervical tissues without lesion(including HPV16 positive and hr-HPVs negative) were obtained from total hysterectomy at the same time with benign tumors. All tissue specimens were diagnosed by pathologists from Guangxi Medical University cancer hospital and prepared 4 μm thick continuous section for staining. All of these samples contained the results of preoperative HPV-DNA typing test, in which178 (82.8%) were infected with HPV16 type, and 37 (17.2%) were infected with non-hr-HPVs (including HPV-negative).

### Immunohistochemical staining and result interpretation

Tissue specimens were fixed with 10% formalin and wrapped with paraffin,

then sequentially sectioned into 4 μm thick slices and roasted for 2 h at 65 ℃, followed by xylene dewaxing, gradient ethanol hydration, boiling water bath repair antigen 40 min, 3% hydrogen peroxide endogenous biotin was blocked for 15 min, endogenous biotin was blocked for 20 min, goat serum was blocked for 30 min, release antibody working fluid(1:100), incubate at 4 ℃ overnight. The next day according to rabbit SP kit instructions, secondary antibody was added, and appropriate time of DAB color rendering was followed by hematoxylin redyeing and gradient ethanol dehydration xylene transparent, neutral resin sealing sheet. The known positive sections were used as positive control while PBS was used as negative control instead of primary antibody.

In dye sections with satisfactory staining, 5 fields were searched at 40 × objective. A total of 100 characteristic cells were counted and their average staining intensity and positivity were determined. Semi-quantitative integral method was used to interpret the staining results: (1)strong staining degree: 0 score for no staining, 1 score for light yellow, 2 score for sandy, 3 score for brown; (2) 0 score for positive cell percentage < 5%, 1 score for 6% ~ 25%, 26% ~ 50% was 2 score, 51% ~ 75% was 3 score, and > 75% was 4 score. Immunohistochemical evaluation Points = staining intensity × percentage of positive cells. Immunohistochemical score 0 is negative(-), namely nuclear-free expression;1 ~ 4 is weakly positive ( +), that is, low nuclear expression; 5 ~ 8 is positive (+ +), 9 ~ 12 is strongly positive (+ + +), that is, high nuclear expression. The dyeing results were evaluated by two pathologists with double-blind method. The same value is the final score, if there is a different value, the third observer was conducted to joint assessment.

### Cell culture

SiHa (HPV-16-positive cervical cell line), C33-A (HPV-negative cervical cell line) and 293FT(Human embryonic kidney cell line) were purchased from Cell Resource Center,IBMS, CAMS/PUMC (Beijing, China) and maintained in Dulbecco’s Modified Eagle’s Medium (Gibco,China) supplemented with 10% Fetal Bovine Serum and 1% penicillin/streptomycin (Gibco, China) at 37 °C and 5% CO2.

### miRNA and mRNA real-time quantitative RT-PCR analysis

Total RNA was extracted from the cell lines in this study using the RNeasy Kit (Qiagen, USA) following the manufacturer’s instructions. To measure miRNA levels, 2 mg total RNA was subjected to analysis using Bulge-LoopTM miRNA qRT-PCR detection kit, and small nucleolar U6 was used as the housekeeping small RNA reference gene (RiboBio, Guangzhou, China). In total, 500 ng total RNA was reversed transcribed using the iScript Reverse Transcription Supermix and measured with Sofast Eva Green Supermix (Bio-Rad, USA) using a Bio-Rad CFX96 Real Time system (Bio-Rad,USA) according to the manufacturer’s instructions. GAPDH was used as a housekeeping gene control for normalization of the expression level of all mRNAs. All primer sequences are listed in Additional file 1: Table S1.

### Modulating miRNAs, mRNAs expression in cell lines

miRNAs levels were modulated by corresponding inhibitor or mimics (RiboBio, Guangzhou, China). miRNA mimics are chemically synthesized double-stranded RNAs, while miRNA inhibitors are chemically synthesized single-stranded RNAs. These agents can be transfected into a variety of cell types. The corresponding controls for the miRNAs inhibitor and mimic were provided in the same kit. After transfection, miRNAs expression was quantitated by qRT-PCR as described above. NF-κB family members and HPV16 E5, E6, E7 overexpression was achieved by transfecting vectors carrying the corresponding genes. First, all genes were amplified from SiHa cell genomic DNA and cloned into the lentiviral vector p2K7neo as previously described. The expression of all genes was driven by the human EF1a promoter. The primers used to PCR amplify these genes are listed in Additional file 1: Table S1. Lentiviral supernatant was collected to infect C33-A cells as described previously. To silence them, shRNAs were constructed using the Block-iT inducible H1 lentiviral system (Invitrogen, USA) according to the manufacturer’sprotocols. shRNAs were first inserted into pENTR/H1/TO vectors, and LR was recombined into pLenti4/BLOCK-iTTM-DEST. Lentiviral supernatant was collected to infect SiHa Cells as described previously. Cells were collected 48 h after lentiviral infections and subjected to quantitatively real time RT-PCR.

## Cells proliferation assay

Cell proliferation assays were performed using the DNA Proliferation in vitro Detection Kit following the manufacturer’s instructions (RiboBio, Guangzhou, China). Cells were sequentially incubated with the thymidine analog5-ethynyl-29-deoxyuridine (EdU) for 3 h, Apollo567 for 30 min, and Hoechst33342 for 30 min. Cell images were captured using a fluorescence microscope (Nikon Eclipse-Ti, Japan). Serial optical sections were captured using NIS-Elements software and projected to provide two-dimensional maximum brightness images. More than 50 cells in three different areas of three independent cultured wells (n = 9) for each sample were counted for EdU-positive staining followed by statistical analysis.

### Annexin V-FITC/PI apoptotic assay

Apoptosis assays were conducted using the Annexin V-FITC/PI Apoptosis Kit following the manufacturer’s instructions (KeyGEN Biotech, Nanking, China)0.10,000 cells were resuspended in 500 ml binding buffer and incubated with Annexin V-FITC and propidium iodide for 15 min. The cell suspensions were then subjected to flow cytometry analysis (FACSCalibur, BD, USA). Each experiment was repeated in triplicate.

### Western blotting assay

Cells were cultivated according to experimental requirements in 6-well plates, and RIPA lysis buffer (EpiZyme) was added with protein phosphatase inhibitor mix (EpiZyme) at a proportion of 1:100. The lysates.

were placed on ice for 30 min and swirled in 6-well plates occasionally.

Then centrifuged at 4 ^◦^C and 12,000 × g for15 min. Supernatant mix with loading buffer (5 ×) and boiled for 5 min at 85 °C. Following measuring the protein concentration of each sample by a BCA protein quantitative kit (Beyotime), equal amounts of protein (25 μg) were resolved on 10% SDS-PAGE gels through electrophoresis (80 V for 3 h) and transferred to PVDF membranes, which were blocked in QuickBlock™ blocking buffer (Beyotime) for 1 h and incubated with primary antibody (concentration according to instructions) overnight at 4 °C. After washing with TBST (EpiZyme) three times, membranes were incubated with secondary antibodies (1:3000) at room temperature for 1 h. We visualized the protein bands with a chemiluminescent ECL assay kit (Beyotime), and images were captured by a Bio-Rad ChemiDoc XRS + image analyzer. The band density was quantified using ImageJ software.

### Construction of double luciferase reporter gene vectors and detection of luciferase activity

Luciferase reporter gene plasmid (pGL3-Basic) and renillaluciferase reporter vector (PRL-TK) were used to study the interaction between c-Rel and the promoter of miR-133a-3p. Binding site sequence and mutant sequence were obtained through PCR and overlap PCR respectively, the primers are shown in Additional file 1: Table S1.These sequences were inserted into PGL-BASIC and constructed recombinant vector.

Double luciferase vector pisCHECK-2 was used to study the interaction between miR-133a-3p and the 3’UTR of *Akt3* or *Rel*. For construct recombinant vector, the 3'UTR sequence of wild-type *Akt3* and *Rel* or the 3’UTR mutant sequence of *Akt3* and *Rel* were chemical synthesized (Additional file 1: Table S2) and inserted into the upstream of HSV TK Promoter of pisCheck-2, respectively. All the sequences contanined XHO1, NOTI1 restriction sites and 200 bp before and after 3'UTR of *Akt3* and *Rel* were synthesized by Rui Bo Xing Ke Biotechnology Company, Beijing.

293FT cells were seeded into 96-well plates with 10^4^ every well and incubated for 24 h. According to the manufacturer’s instruction, 293FT cells were transiently co-transfected with 200 ng *Rel* OE or p2k7 together with 200 ng miR-133a or miR-133a mt plasmids with 200 ng pRL-TK, while 0.5ul miR-32p-5p mimics together with 100 ng recombinant pisCHECK-2 vector of *Akt3* and *Rel* using Lipofectamine 3000 (Thermo Fisher Scientific; Catalog Numbers:L3000015).After 48 h, firefly and Renilla luciferase activities were detected through Promege kit Dual-Luciferase Reporter Assay Sestem(E1960) according with manufacturer’s instructions. The luciferase activity and Renilla luciferase activity of firefly were measured respectively in all groups first by LB941 Microporous plate luminescence instrument (Guangzhou Yunxing Scientific Equipment Company, Software Version MikroWn 2000 Version 1.05).Then calculated the ratio of firefly luciferase activity to Renilla luciferase activity through dividing the luciferase activity of firefly by the luciferase activity of Renilla.

### Statistical analysis

The mean ± standard deviation from three independently repeated experiments were calculated. Comparisons of the different treatment groups were performed with Student's t-test and one-way analysis of variance (ANOVA). p-values < 0.05 were considered statistically significant difference.

## Results

### The prediction of HPV16rmiR-TF

According to the results of previous study [5], two groups of miRNAs were proved to be associated with HPV16. The first group of miRNAs including mir-133 family(miR-133a-3p, miR-133a-5p, miR-133b) and miR-196a-5p; the second group of miRNAs (mir-379 ~ 369 cluster), including miR-154-5p, miR-329-3p/5p, miR-382-5p, miR-495-3p, miR-299-5p, miR-376b-3p, miR-379-5p, miR-369-5p, were located in 14q32.31. These HPV16-related miRNAs were down-regulated in different stage of HPV16 infected cervical tissues in turn. TRANSFAC and JASPAR were used for matching and prediction transcription factors of every miRNA precursor mentioned above, the results that can be predicted by both methods were selected as candidate transcription factors of single miRNA. The candidate transcription factors of each miRNA were showed in Additional file 1: table S2. Among them, the highest frequency of transcription factors were NF-κB family members, including c-Rel, p65 and p50, considered as the main HPV16rmiR-TFs. In addition, after target genes prediction through a series of databases such as TargetScan, miRDB and miRanda, the miRNAs, transcription factors and target genes were integrated into TFs-miRNAs-target genes networks (Additional file 1: Fig. S1 and Fig. S2). Macroscopically, a great numbers of members involve in a huge network of HPV16 infection.

### The nuclear expression characteristic of main HPV16rmiR-TFs(NF-κB family members c-Rel, p65 and p50)on HPV16 infected cervix tissues

**Fig. 1:**
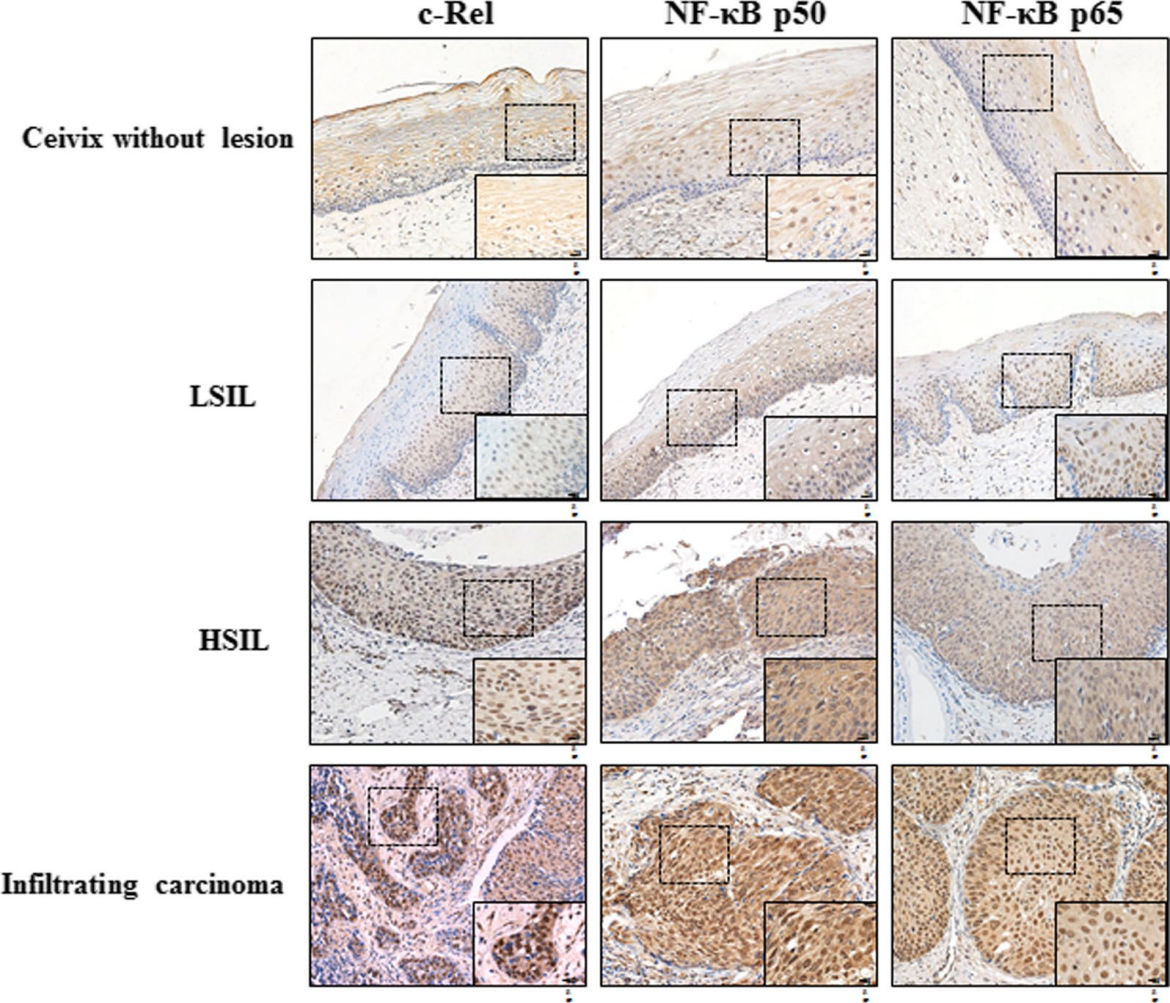
Representative images of immunohistochemical detection for NF-κB family (c-Rel,p50 and p65) in cervical tissues (Original images magnification 200 ×, Magnified images magnification 400×

Immunohistochemical staining on HPV16 infected cervix samples showed that c-Rel, p65 and p50 were located in nucleus and cytoplasm. Since most NF-κB expression in cytoplasm were mainly in an inactive state [11], this study considered and determined their staining status in nucleus only. The immunohistochemical examples of c-Rel, p65, p50 are shown in Fig. 1 while the expression analysis of c-Rel, p65 and p50 are shown in Table 1.The expression rate of c-Rel increased with the aggravation of cervical lesions. There were statistically significant differences in overall expression of groups (χ^2^ = 21.953, P < 0.001). Among them, the positive expression rate in infiltrating carcinoma(69%) and HSIL tissues(76.9%) was significantly higher than that in cervical tissues without lesion(35.3%) and LSIL tissues(54.5%). There were no significant differences between whether HSIL and infiltrating carcinoma groups, or cervical tissue without lesion and LSIL groups (χ^2^ = 6.027, P = 0.110; χ^2^ = 2.956, P = 0.415 respectively).

**Table 1:** Expression of c-Rel, p50 and p65 in different cervical tissues

Category		Cervix without lesion	LSIL	HSIL	invasive carcinoma	χ2	P
c-Rel		51	22	26	116		
	−	33	10	6	36		
	+	6	5	3	35		
	+ +	8	5	8	28	19.873	< 0.001
	+ + +	4	2	9	17		
	Positive rate (%)	35.3	54.5	76.9	69		
NF-ĸB p65	−	30	10	13	40		
	+	11	9	4	51		
	+ +	6	1	5	16	16.356	0.011
	+ + +	4	2	4	9		
	Positive rate(%)	41.2	54.5	50	65.5		
NF-ĸB p50	−	31	11	9	31		
	+	14	5	4	62		
	+ +	5	5	6	16	17.205	0.001
	+ + +	1	1	7	7		
	Positive rate(%)	39.2	50	65.4	73		

p50 mainly expressed in HSIL and infiltrating carcinoma tissues. The positive rate in HSIL(65.4%) and infiltrating carcinoma tissues(73%) were significantly higher than that in cervical tissue without lesion(39.2%) and LSIL tissues(50%). Moreover, p50 positive expression rate in cervical invasive carcinoma group was significantly higher than that in HSIL group, the difference was statistically significant(χ^2^ = 17.672, P = 0.001), while there was no significant difference between cervical tissue without lesion and LSIL groups(χ2 = 3.027, P = 0.372). The overall expression was statistically significant difference among all groups (χ^2^ = 17.205, P = 0.001).

The overall expression of p65 was difference in all groups with statistical significantly(χ2 = 16.356, P = 0.011). Among them, p65 positive expression rate in LSIL(50%), HSIL(54.5%) or cervical tissue without lesion(41.2%) group has no significant difference, which all significant lower than that in infiltrating carcinoma tissues(65.5%)(χ^2^ = 9.905, P = 0.019; χ ^2^ = 7.697, P = 0.048; χ^2^ = 8.473, P = 0.031 respectively).

### The relationship between nucleus expression of c-Rel, p65, p50 and the stage of HPV16 infection

The nucleus expression characteristic of c-Rel, p65, p50 indicate that they may play roles respectively in different stage of cervical lesion. To our knowledge, the clinical outcome of patients with LSIL and HSIL are significantly different according to series of related studies [12], Meanwhile, cervical cancer screening-related molecular markers such as P16 are often expressed significantly differently between LSIL and HSIL tissues [13]. Therefore, whether from clinical or molecular pathological perspectives, LSIL/HSIL should be a distinct and appropriate demarcation point to distinguish HPV16 early infection from late infection.

Therefore, the 215 cervical samples in our study were divided into three groups: (1) Negative control: hr-HPVs negative (including HPV negative cervix without lesions); (2) Early infection: including HPV16 positive tissues with LSIL and cervix which has not yet lesions; (3) Advanced infection: HPV16 positive HSIL and infiltrating cervical carcinoma tissues. The difference of these transcription factors expression in these groups are shown in Table 2.The positive expression rates of c-Rel and p65 in early infection group were significantly higher than those in control group (52.8% vs 29.7%, 61.2% vs 29.7%, χ^2 ^= 5.69310.019; P = 0.017 0.007, respectively). Meanwhile, in advanced infection group, c–Rel and p50 positive rate were significantly higher than those in the early infection group (73.2% vs 52.8%, 71.8% vs 47.2%; χ^2^ = 6.229, 8.014; P = 0.044, 0.018,respectively).

**Table 2:** Expression of c-Rel, p50 and p65 in different stage of HPV16

	Negative Control (n = 37)	Early Infection 36)			Late Infection(n = 142)		
	hr-HPVs(-) without lesion	HPV16( +) cervix without lesion /LSIL	χ^2^	P	HPV16( +) HSIL/infiltrating carcinoma	χ^2^	P
c-Rel							
Negative (%)	26 (70.3)	17 (47.2)	5.693	0.017	38(26.8)	6.229	0.044
Low (%)	6 (16.2)	5 (13.9)			38 (26.8)		
High (%)	5 (13.5)	14 (38.9)			66 (46.4)		
NF-ĸB p65							
negative (%)	26 (70.3)	14 (38.8)	10.019	0.007	53 (37.4)	1.033	0.597
Low (%)	9 (24.3)	11 (30.6)			55 (38.7)		
High (%)	2 (5.4)	11 (30.6)			34 (23.9)		
NF-ĸB p50							
Negative (%)	23 (62.2)	19 (52.8)	0.753	0.686	40 (28.2)	8.014	0.018
Low (%)	9 (24.3)	10 (27.8)			66 (46.4)		
High (%)	5 (13.5)	7 (19.4)			36 (25.4)		

Further Logistic regression analysis showed that c–Rel and p65 are related to early infection of HPV16, their nuclear expressions are positively correlated with the occurence of early HPV16 infection with statistically significant (OR = 4.282, 8.214; 95% confidence interval: 1.303–14.078, 1.980–42.696; P < 0.05); while c–Rel and p50 are related to late infection of HPV16, their nuclear expressions are positively correlated with the development of HPV16 infection with statistically significant (OR = 2.449, 3.978; 95% confidence interval: 1.154–5.197, 1.595–9.924; P < 0.05) (Table 3).

**Table 3:** Logistic regression analysis of nuclear expression of c-Rel, p50,p65 and development of cervical cancer

	Negative control	Early infection		Late infection
	hr-HPVs(−)	HPV16( +)		HPV16( +)
	Without lesion/LSIL	Without lesion/LSIL		HSIL/carcinoma
N	37	36		142
c-Rel				
P	0.017		0.028	
OR	4.282		3.400	
95% confidence interval	1.303–14.078		1.139–10.151	
p65				
P	0.015		0.659	
OR	8.214		0.816	
95% confidence interval	1.980–42.696		0.332–2.007	
p50				
P	0.799		0.012	
OR	1.076		3.978	
95% confidence interval	0.348–3.330		1.595–9.924	

Thus it can be seen that p65 and p50 acts as related risk factor for early and late HPV16 infection respectively, which might play tumorigenesis or progression role in different periods. While c-Rel, which gradually increased during the whole evolution from normal cervix to cervical cancer after HPV16 infection and significantly correlated with the degree of cervical lesions, is a risk factor throughout the occurrence and development of cervical cancer during the whole process of HPV16 infection, involved in the whole transformation process induced by HPV16.

### c-Rel, p65 and p50 act as mediators which link the HPV16 and two groups of miRNAs

Thus NF-κB family c-Rel, p65, p50 were predicted as probable HPV16rmiR-TFs and have been shown increased at different stage of HPV16 infection, they may act as mediators which linking the HPV16 infection and the two groups of miRNAs.

**Fig. 2:**
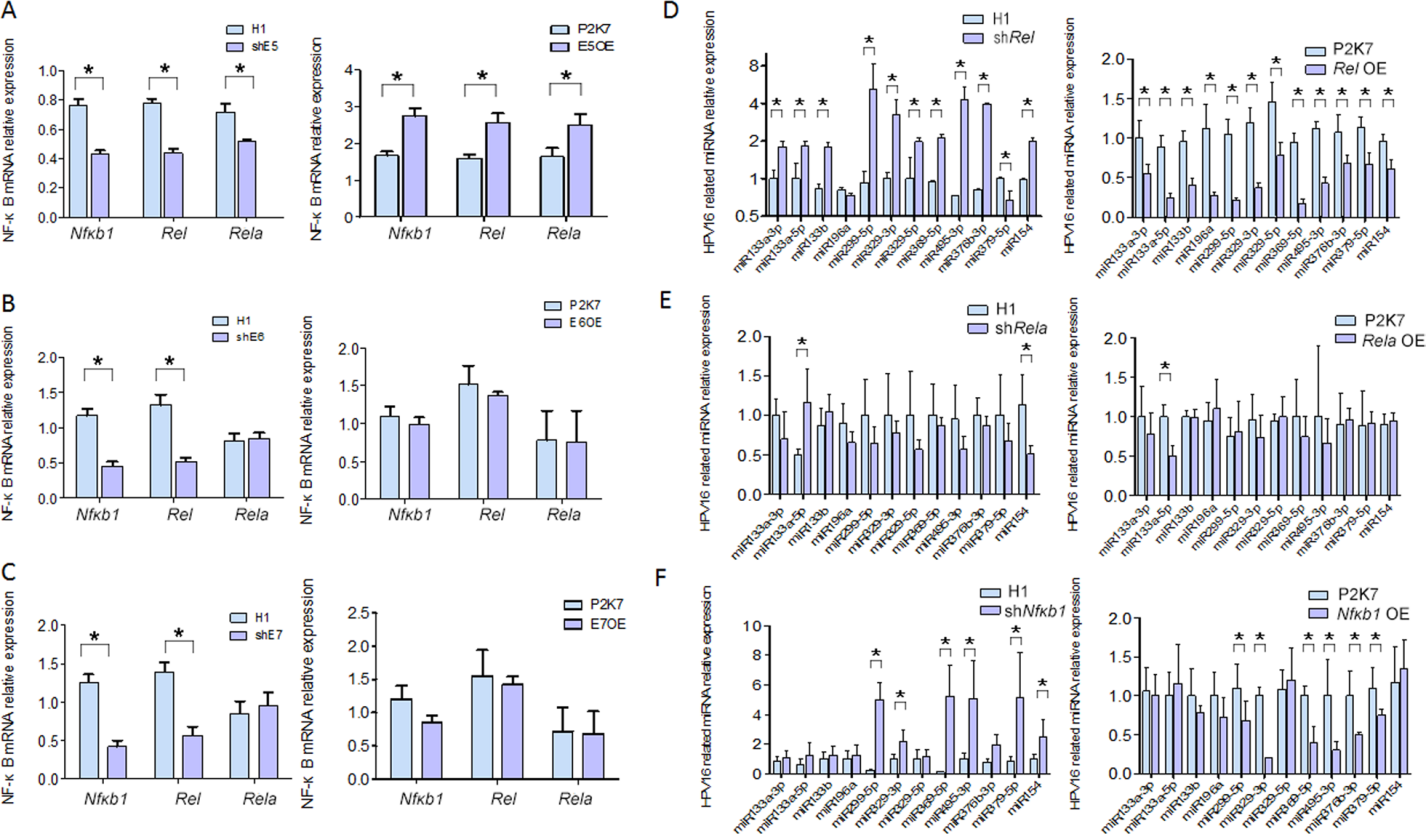
c-Rel, p65 and p50 link the HPV16 oncoprotein and two groups of HPV16rmiRNAs. A: *Rel*, *Nfκb1* and *Rela* were regulated by HPV16 E5, they were increased by the increase of E5 and decreased by the decrease of E5; B: *Rel*, *Nfκb1* and *Rela* were not regulated by HPV16 E6, Rel and *Nfκb1* were decreased with the decrease of E6,but they were not changed when E6 overexpression; C: *Rel*, *Nfκb1* and *Rela* were not regulated by HPV16 E7, Rel and *Nfκb1* were decreased with the decrease of E7,but they were not changed when E7 overexpression. D: HPV16rmiRNAs were regulated by c-Rel, almost all these miRNAs increased when the sh*Rel* transfected in SiHa cells and decreased when the *Rel* OE transfected in C33-A cells; E: p65 regulate miR-133a-5p which increased when the sh*Rela* transfected in SiHa cells and decreased when the *Rela* OE transfected in C33-A cells; F: miR-299-5p,miR-329-3p, miR-369-5p, miR-495-3p, miR-376b-3p, miR-379-5p were modulated by p50,they increased when the sh*NFκb1* transfected in SiHa cells and decrease when the *NFκb1* OE transfected in C33-A cells. *:P < 0.05

To test this hypothesis, we first demonstrated the direct relationship between HPV16 early genes and the expression of c-Rel, p65, p50 coding genes *Rel*,*Rela* and *Nfκb1*. Overexpression and shRNA vectors were constructed respectively to modulate HPV16 E5, E6,and E7 which expressed separately from the corresponding ectopic vector(named E5OE, E6OE, E7OE; shE5, shE6, shE7 respectively). P2K7 and H1 were transfected as empty control vectors (negative control)of overexpression and shRNA vector respectively. The expression of HPV16 E5, E6,and E7 after transfection are shown in Additional file 1: Fig. S3. After that, the effect on *Rel*,*Rela* and *Nfκb1* expression were examined by qRT-PCR independently (Fig. 2). When SiHa cells were transfected by shE5 vector, the expression of *Rel*,*Rela* and *Nfκb1* were all decreased compared with negative control(All P values are less than 0.05), while when E5OE vectors were transfected into C33-A cells, the expression of *Rel*,*Rela* and *Nfκb1* were all increased compared with negative control(All P values are less than 0.05). Differently, although shE6 and shE7 vectors infection could decrease *Rel* and *Nfκb1* expression of SiHa cells(these two P values are less than 0.05), E6OE and E7OE vector could not correspondingly increase the *Rel* and *Nfκb1* expression in C33-A cells. So the data showed that *Rel*,*Rela* and *Nfκb1* were regulated by HPV16 E5, but not sure be modulated by HPV16 E6 and E7.

The direct relationship between *Rel*,*Rela* and *Nfκb1* and the two groups of miRNAs were also needed to be demonstrated. So we then constructed overexpression vectors and shRNA vectors to modulate *Rel*,*Rela* and *Nfκb1* transcriptional expression (named *Rel* OE, *Rela* OE, *Nfκb1* OE, sh*Rel*, sh *Rela*, sh*Nfκb1* respectively), The expression of *Rel*,*Rela* and *Nfκb1* after transfection are shown in Additional file 1: Fig. S4, afterward, the effect on each miRNA expression level were evaluated by qRT-PCR independently. The data showed that almost all the miRNAs (including miR-329-3p/5p, miR-382-5p, miR-495-3p, miR-299-5p, miR-376b-3p, miR-379-5p, miR-369-5p) were regulated by *Rel* (Their P values are less than 0.05), the first group miRNA member miR-133a-5p was modulated by *Rela* (P < 0.05), while most members of the second group of miRNAs (including miR-299-5p,miR-329-3p, miR-369-5p, miR-495-3p, miR-376b-3p, miR-379-5p) were modulated by *Nfκb1* (Their P values are less than 0.05) (Fig. 2).

###  c-Rel affects the occurrence and progression of cervical cancer during whole HPV16 infection stage through mir-133 family and miR133a-3p–modulated mir-379–369 cluster

Unlike p65 and p50, c-Rel act as a risk factor throughout the occurrence and development of cervical cancer during the whole process of HPV16 infection according data above. Consistently, after modulate the expression of c-Rel, not only group 1 mir-133 family, but also the majority members of mir-379-369 cluster were affected as well, while p65 and p50 increases the risk of cervical cancer incidence or development in early or late stages of HPV16 infection by affecting only miR-133a-5p or mir-379-369 cluster corresponding respectively.

**Fig. 3:**
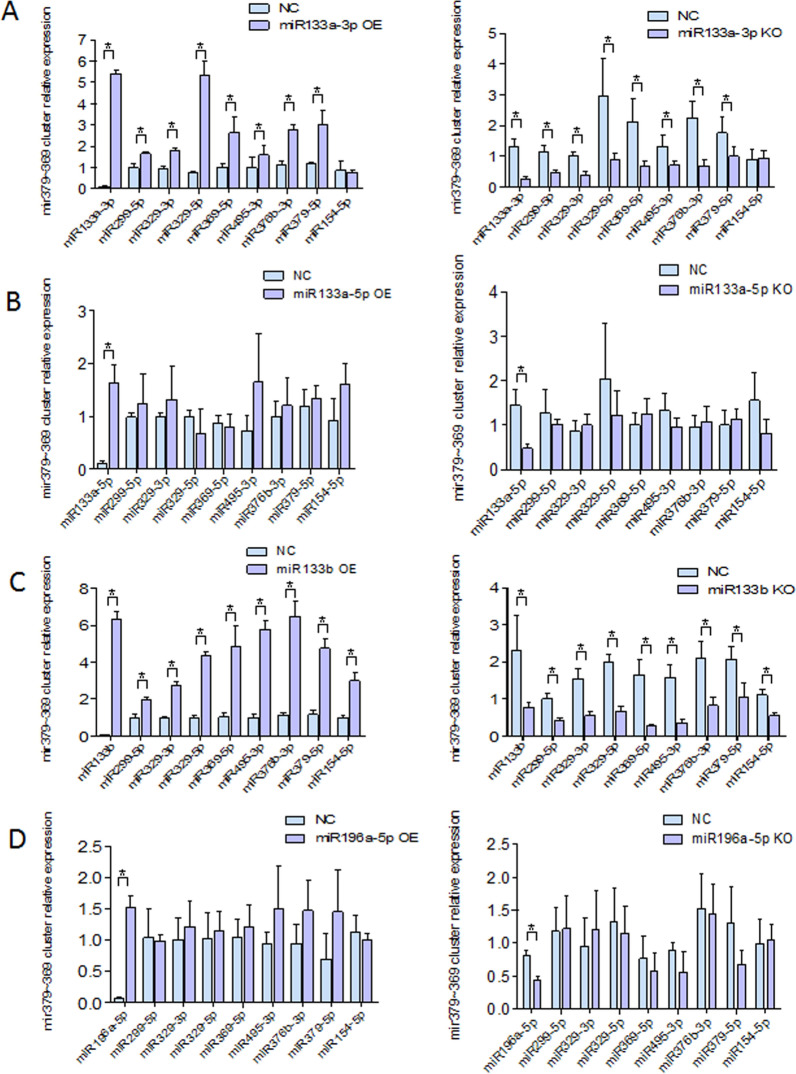
The regulation relationship between two groups of HPV16rmiRNAs. A: miR-133a-3p regulates the group 2 miRNAs, miR-299-5p,miR-329-3p, miR-369-5p, miR-495-3p, miR-376b-3p, miR-379-5p increased with the increase of miR-133a-3p and decreased with the decrease of miR-133a-3p. B:The group 2 miRNAs are not affected by miR-133a-5p; C: miR-133b regulates the group 2 miRNAs, miR-299-5p,miR-329-3p, miR-369-5p, miR-495-3p, miR-376b-3p, miR-379-5p and miR-154-5p increased with the increase of miR-133b and decreased with the decrease of miR-133b; D: The group 2 miRNAs are not affected by miR-196a.*:P < 0.05

Although these results revealed parts of relationship among HPV16 infection, HPV16rmiR-TFs, two groups of miRNAs and the progression of cervical lesions, the exact mechanisms in which are not yet entirely clear. Due to the two sets of miRNAs change steply in HPV16 cervical lesions, there may be regulatory relationship between them, especially the cumulative effect of the first group miRNAs may affect the expression and function of the second group miRNAs. So to test this hypothesis, when mir-133 family and miR-196a were modulated through corresponding miRNA inhibitors or miRNA mimics transfected, the second group miRNAs (mir-379–369 cluster) expression were tested. The results showed that miR-133a-3p and miR-133b are the main mediators of mir-379–369 cluster members, while the other two miRNAs (miR-196a and miR-133-5p) of the first group miRNAs had no significant effect on the second group miRNAs (mir-379–369 cluster members) (Fig. 3).

### HPV16 E5, *c-Rel* and miR-133a-3p all affect cell proliferation, apoptosis, migration, invasion and autophagy

The potential cellular function of HPV16 E5, c-Rel and miR-133a-3p were investigated by examining the effect on cell proliferation, apoptosis, migration, invasion and autophagy in this study.

**Fig. 4:**
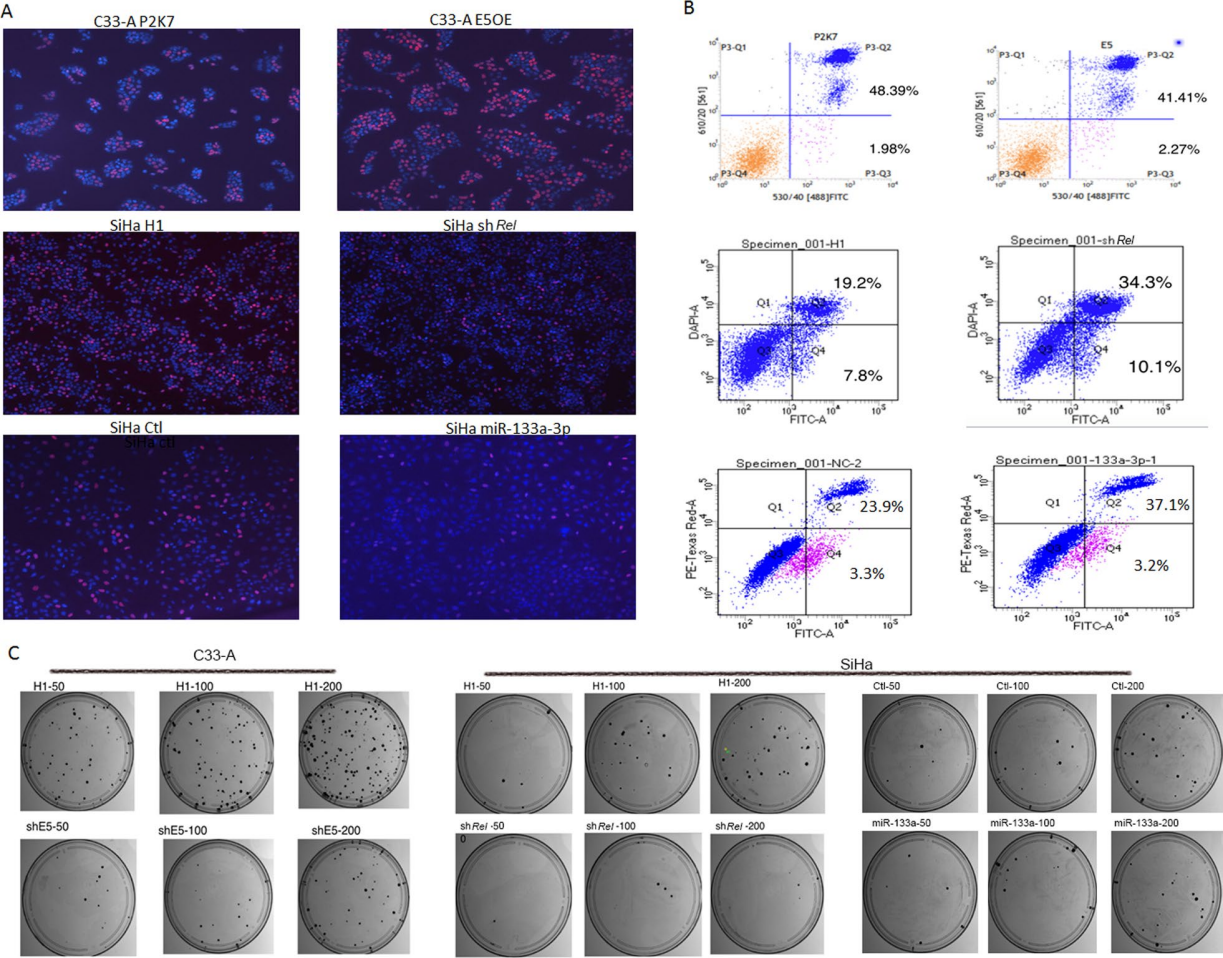
HPV16 E5, c-Rel and miR-133a-3p all affect cell proliferation, apoptosis, migration, invasion and autophagy. A: EdU staining indicating C33-A cell proliferation increases when HPV16 E5 is overexpressed; EdU staining indicating SiHa cell proliferation decreases when *Rel* is silenced and when miR-133a-3p is overexpressed; B: flow cytometry analysis of annexin V and propidium iodide-stained C33-A cells transfected with HPV16 E5 OE or SiHa cells treated *Rel* shRNA,miR-133a-3p mimics; C:The colony-formation ability of SiHa cells with shHPV16E5,sh*Rel* and miR-133a-3p mimics compared with negative control from 50 to 200 cell inoculation densities, the colony-formation ability have all been reduced

The effect on cell proliferation was determined by conducting EdU assays. The results showed that in C33-A cells, transfer the HPV16 E5 expression increased the percentage of EdU incorporated cells to (71.67 ± 3.712)% compared with the control, which exhibited (46.67 ± 4.41)% incorporation(P < 0.05). However, EdU incorporation decreased to (12.67 ± 0.9)% when *Rel* was downregulatedd and to (10. 8 ± 0.4726)% when miR-133a-3p was overexpressed compared with (25.05 ± 0. 865)% and (21.93 ± 0.9753)% respectively in the control in SiHa cells(All P values are less than 0.05) (Fig. 4A).

**Fig. 5:**
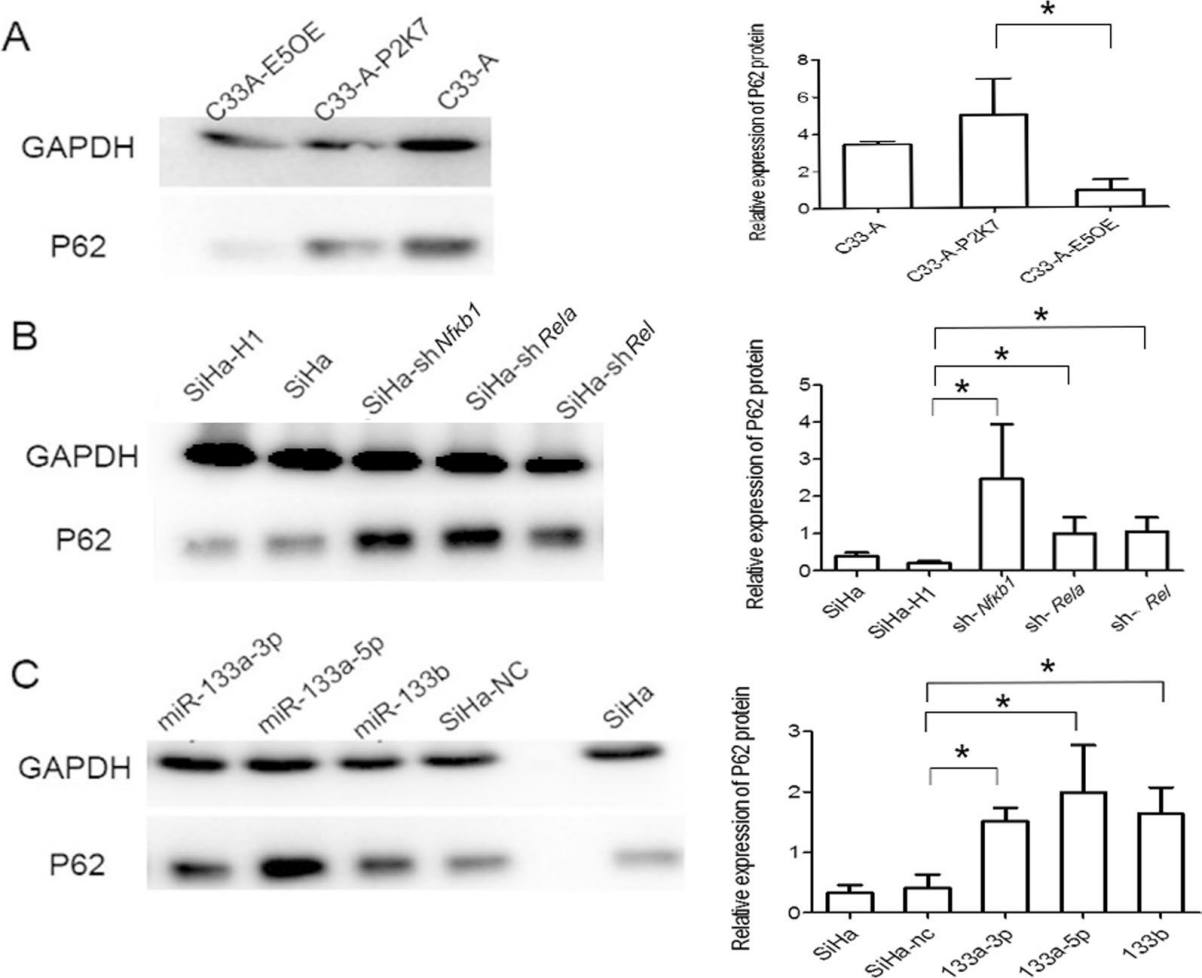
Western blotting was utilized to detect the protein relative expression of early autophagy proteins P62 in cervical cancer cell. A: P62 relative expression in C33A with HPV16 E5OE VS. P2K7 vector. The bar chart indicate that P62 expression decrease when HPV16 E5 overexpression; B: P62 relative expression in SiHa with *Rel* shRNA,*Nfκb1* shRNA,*Rela* shRNA VS.H1 vector. The bar chart indicate that P62 expression increase when *Rel*, *Nfκb1* and *Rela* knockdown; C: P62 expression in SiHa with miR-133 mimics VS negative control. The bar chart indicate that P62 expression increase when miR-133 overexpression.* P < 0.05

To evaluate the effect of HPV16 E5, c-Rel and miR-133a-3p on cell apoptosis, we performed Annexin V/propidium iodide (PI) assay. Annexin V + /P stained cells represent early apoptotic cells, whereas Annexin V + /PI + stained cells represent late apoptotic cells or necrotic cells. Transferring HPV16 E5 to C33-A cells significantly decreased late apoptotic percentage (48.6% ± 0.729% vs. 41.07% ± 0.415%, P < 0.05), whereas *Rel* down-regulation and miR-133a-3p up-regulation increased the SiHa cell apoptosis (early apoptotic cells with P < 0.05: 7.367% ± 0.219% vs. 10.34% ± 0.307%, late apoptotic cells with P < 0.05: 18.77% ± 0.589% vs. 32.7% ± 0.923%; early apoptotic cells with P > 0.05: 3. 293% ± 0.0176% vs. 3.210% ± 0.026%, late apoptotic cells with P < 0.05: 23.57% ± 1.053% vs. 35.53% ± 0.845% respectively) (Fig. 4B). In addition, the colony-formation ability of cells were compared in terms of plate colony assay. The results showed that colony-formation ability of SiHa cells with shHPV16E5 were reduced compared with SiHa cells with empty H1 vector; While in SiHa cells with sh*Rel* compared with H1 vector, or in SiHa cells with miR-133a-3p mimics compared with negative control sequence, the colony-formation ability have all been reduced (Fig. 4C);

After that, the effect of HPV16 E5, c-Rel and miR-133a-3p on early autophagy were tested through P62 protein western blotting assay. The results showed that in C33-A cells, transfer the HPV16 E5 OE vector decreased the P62 expression. In contrast, when *Rel* was inhibited and miR-133a-3p was up-regulated, the protein level of P62 was significantly increase (Fig. 5). It means that HPV16 E5, c-Rel and miR-133a-3p all affect the ability of cell autophagy, HPV16 E5 and c-Rel can improve early autophagy capacity while miR-133a-3p which down-regulated by them can weaken autophagy capacity.

### *c-Rel* affects miR133a-3p and subsequent mir-379-369 cluster with a positive feedback way

**Fig. 6:**
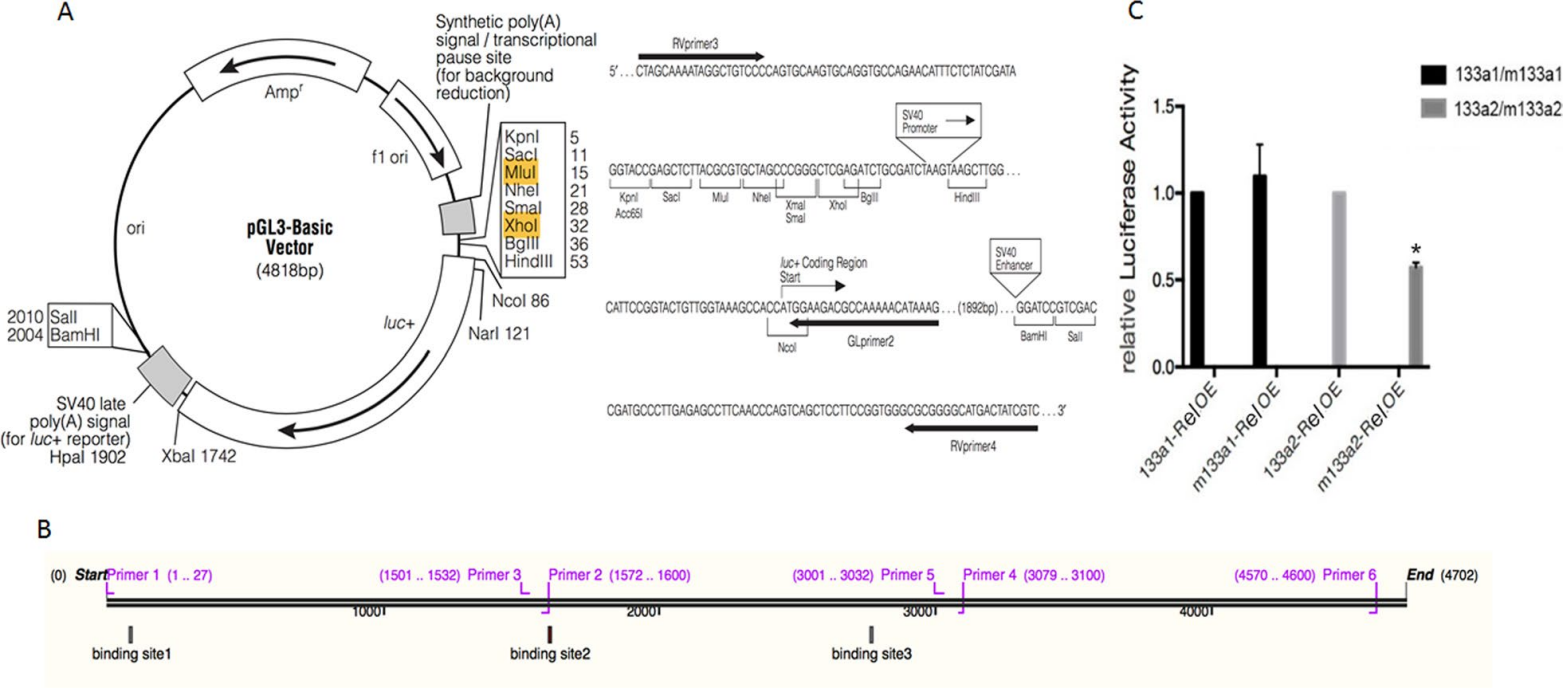
*Rel* binds directly to the promoter sequence of mir-133a precursor. A:The map of pGL3-Basic vector and the insert position in it; B: The binding site of *Rel* on mir-133a precursor. Among them, two mutant site(miR-133a2mt and miR-133a3mt) were located in bingding site 2; C:The activity luciferase of miR-133a2 VS. miR-133a2mt is 0.516, which showed that *Rel* can bind directly to the promoter sequence of mir-133a precursor binding site 2.* P < 0.05

As a transcription factor that functions throughout the whole process of HPV16 infection, *Rel* was predicted binding directly to the promoter sequence of mir-133a precursor through JASPAR. To verify the prediction, wild-type mir-133a promoter(wt) sequence or mutant-type mir-133a promoter(mt) sequence were inserted into the firefly luciferase reporter gene upstream MCS of dual-luciferase vector pGL3-Basic respectively (named miR-133a wt and miR-133a mt) (Additional file 1: Fig. S3), *Rel* overexpression vector plus with miR-133a wt or miR-133a mt pGL3-Basic vectors, renillaluciferase reporter vector (PRL-TK) were co-transfected to 293FT cells, a significant reduction of luciferase activity was observed in miR-133a wt compared with miR-133a mt (P < 0.05), it confirmed that the promoter sequence of mir-133a precursor is bound to transcription factor *Rel*.(Fig. 6).

**Fig. 7:**
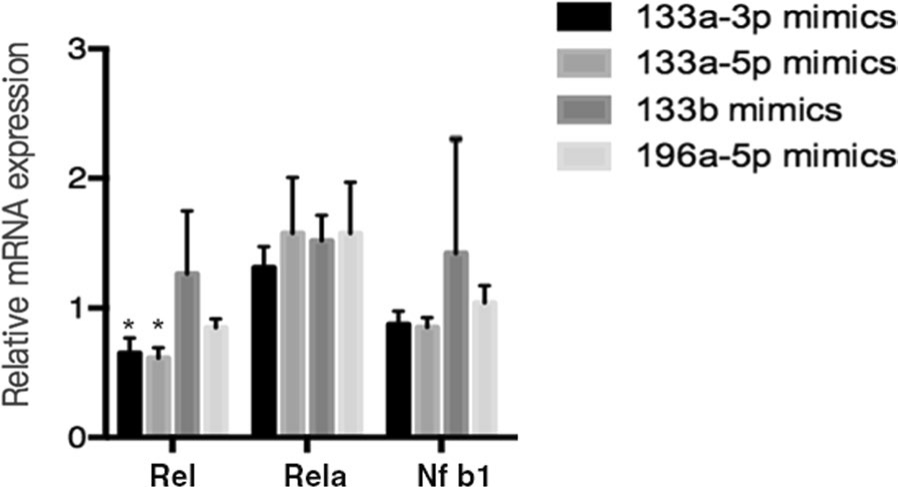
miR-133a affects the *Rel* expression in SiHa cells. When miR-133a mimics transfected to SiHa cells, the *Rel* expression decreased significantly.*:P < 0.05

Interestingly, the inhibition of miR133a-3p by *Rel* seems to be strengthened in a positive feedback manner. Briefly, after overexpress the expression of miR-133a, the *Rel* expression was found decreased accordingly (P < 0.05, Fig. 7). It suggests that there exists mutual regulation between miR-133a and *Rel*. *Rel* not only regulate miR133a-3p by acting directly on its precursor promoter, but also can be affected by the mature miR133a-3p directly or indirectly in turn. While through miR133a-3p and this feedback loop, the mir-379-369 cluster members down regulated followed. This founding also explain why c-Rel plays roles on HPV16 infection in cervical lesions from early to late stage.

### miR133a-3p affects c-Rel in turn through miR329-5p which targets at *Akt3* and *Rel* itself to form the feedback loop

**Fig. 8:**
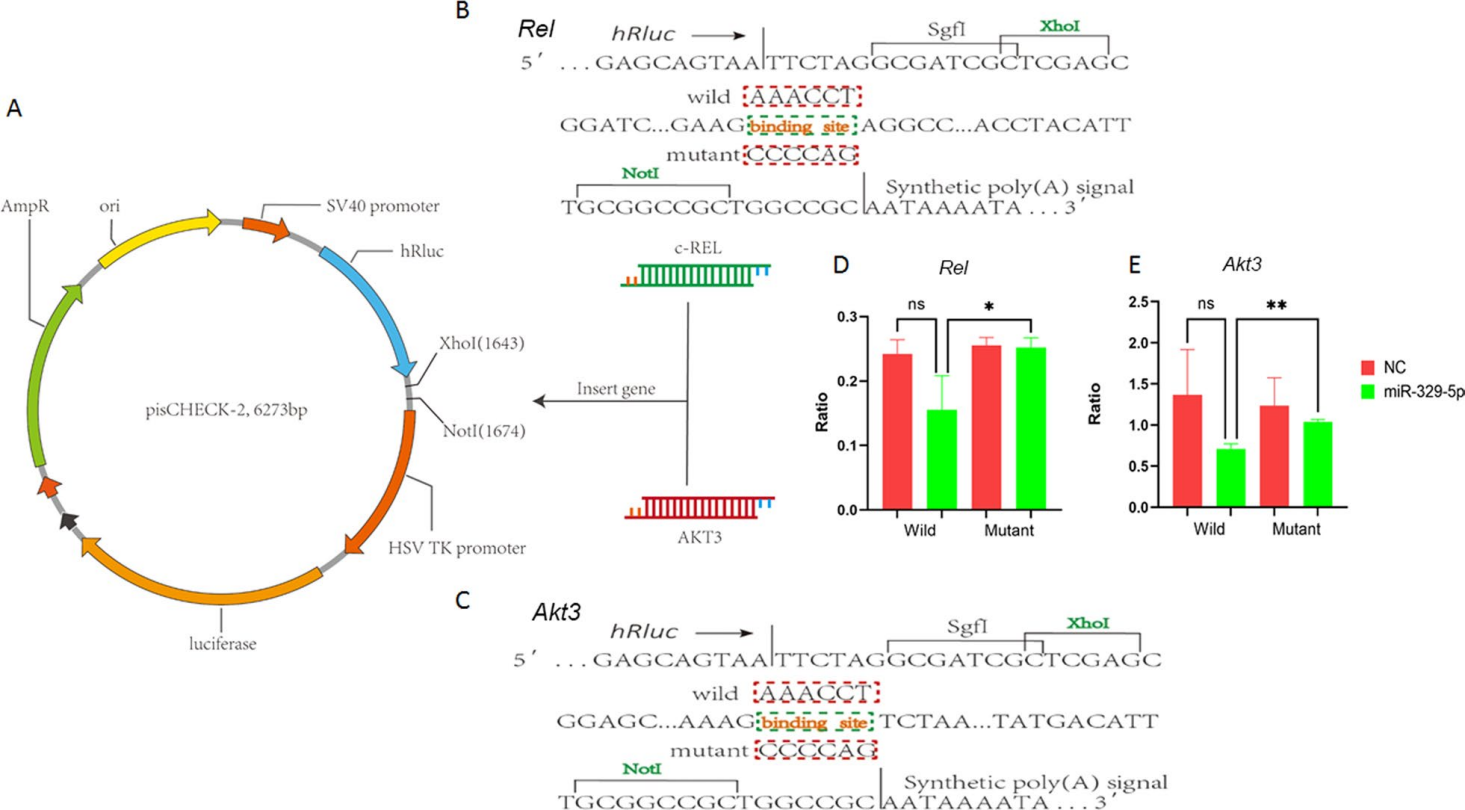
miR329-5p can combine 3’UTR of *Rel* and *Akt3*.A: The map of pisCHECK-2 vector and the insert position in it. B: The *Rel* 3’UTR binding site of miR329-5p; C: The *Akt3* 3’UTR binding site of miR329-5p; D: The activity luciferase of *Rel* wt and *Rel* mt; E: The activity luciferase of *Akt3* wt and *Akt3* mt.*:P < 0.05

About the positive feedback loop, although it has been demonstrated that *Rel* can combine at the precursor promoter of miR133a-3p to regulate it, the mechanisms by which miR133a-3p affects c-Rel in turn remained unknown. So through TargetScan, HPV16-related miRNAs’ target genes were predicted. Among the predicted target genes, miR-133a-3p is not found to bind *Rel* 3’UTR directly, so it can be considered that the regulation of c-Rel by miR-133a-3p is not direct. In spite of it, the second group of HPV16 related miRNAs mir-379-369 cluster member miR329-5p which be regulated by miR-133a-3p, can be predicted combine 3’UTR of *Rel* and *Akt3* (a known positive regulator of c-Rel)(14, 15). Therefore, the 3’UTR wild sequence or mutant sequence of *Akt3* and *Rel* were inserted into the upstream of HSV TK promoter of dual-luciferase vector pisCHECK-2 respectively, which named *Akt3* mt, *Akt3* wt, *Rel* mt, *Rel* wt respectively. Compared with *Akt3* 3'UTR mt, when miR-329-5p mimics and *Akt3* 3'UTR wt were co-transfected into 293FT cells, the enzyme activity fluorescein is observed significantly reduced (P < 0.05), confirming that miR-329-5p binds to 3'UTR of *Akt3*. In the same way, it can be observed that compared with *Rel* 3’UTR mt, the luciferase activity of *Rel* 3'UTR wt is significantly reduced (P < 0.05), which also confirming that miR-329-5p binds to 3'UTR of *Rel*. So both *Akt3* and *Rel* are the target genes of miR-329-5p in the miR379 ~ 369 cluster (Fig. 7). These results all indicate that miR-133a-3p mediated the positive feedback loop of c-Rel through the member of miR379 ~ 369 cluster miR-329-5p which targets *Akt3* and *Rel*. (Fig. 8).

## Discussion

High-risk HPVs are highly associated with many types of cancer, especially cervical cancer. Here we began our studies on the basis of pervious founding about two groups of miRNAs which related to HPV16 infection of cervical tissues. These miRNAs demonstrate stepwise down-regulation characteristic from HPV-free normal cervix samples to HPV16-infected cervix samples, or exclusively down-regulated when the cervix is transformed to a cancerous state. That was the first report demonstrating a stepwise down-regulation of miRNAs in cervical cancer development. In addition, we also demonstrated for the first time that the HPV16 early gene E5 specifically reduces miR-196a involved in previous study [5].

The aberrant expression of this two groups of miRNAs, might gradually transform the normal cervix to a cancerous state in the case of HPV16 infection, whose transcription factors were predicted NF-κB family mainly. According to many studies in this area, NF-κB family was confirmed to be interfered by HPV infection [16]. However, most founding of these studies focus on the regulation of HPV-16 E6 and E7 on NF-κB expression, which always present conflicting data whether HPV-16 E6 and E7 stimulate or suppress NF-κB activation [10, 17]. Similar to this, we also concluded that the effects of HPV16 E6 and E7 on NF-κB were not exact through this study. But HPV16 E5 was found to precise regulation effects on NF-κB family c-Rel, p65, p50 through cell model experiment in this study. To our knowledge, this is the first time demonstrating the regulatory relationship between HPV16 E5 and NF-κB family members c-Rel, p65, p50 (transcription factors of these HPV16 related miRNAs) since E5 was reported to reduce miR-196a.

NF-KB family has been confirmed to be involved in the development and progression of many cancers by interacting with multiple miRNAs as transcription factors or downstream target genes [18–20], or even as both transcription factors and downstream target genes of miRNAs to form feedback loops [21, 22]. Not only through bioinformatics predictions, the regulating function of NF-κB family member c-Rel, p65, p50 as transcription factor respectively on the miRNAs above was verified at the first time in this study. Briefly, the first group miRNA member miR-133a-5p was modulated by p65, while most members of the second group of miRNAs (including miR-299-5p, miR-329-3p, miR-369-5p, miR-495-3p, miR-376b-3p, miR-379-5p, miR-154) were modulated by p50, and almost all these miRNAs(including the first and the second group) were regulated by c-Rel.

The regulatory effects of c-Rel, p65 and p50 on miRNAs at different stages of HPV16 infection were also corroborated by the expression characteristics of tissue samples. They all show some degrees of nuclear expression in progression of cervical lesions, which is similar to previous reports [23, 24]. In particular when HPV infection was divided into stages according to the clinicopathological and molecular characteristics of cervical lesions, c-Rel, p65 and p50 were found to show specific associations at corresponding periods of HPV16 infection, respectively. Briefly, p65 which regulates one of member of the first group of miRNA miR-133a-5p, is associated with early HPV16 infection; p50 which regulates most members of the second group of miRNAs, is associated with late HPV16 infection; undoubtedly, c-Rel which regulates almost all members of the two groups of miRNAs, is associated with the whole stages of HPV16 infection. Therefore, in addition to our previous research, a regulating network composed of HPV16 E5, three important transcription factors and these two groups of miRNAs was described in this study, which is closely involved in the pathological changes caused by HPV16 infection. The specific functions of this network have also been proved by the studies about the effects of HPV16 E5, c-Rel and miR-133a-3p on cell proliferation, apoptosis, and autophagy and so on. All of these important members of the network, as interrelated components, participate in the malignant behavior of the cell.

In subsequently, the most striking results about the regulating mechanism of this network were observed, which can explain the reason that c-Rel play roles in the whole process of HPV16 infection by regulating almost all these miRNAs. Although both c-Rel and p65 were found to suppress some miRNAs expression of the first group, the most important difference between them is whether there is an inhibited effect on miR-133a-3p and miR-133b, which were found to link the second group mir-379 ~ 369 cluster with further co-regulation on its members. This shows that c-Rel can down-regulate mir-379 ~ 369 by inhibiting miR-133a-3p and miR-133b. In addition, c-Rel and miR-133a-3p can form a feedback loop which leading to the inhibition of c-Rel on miR-133a-3p and even downstream mir-379 ~ 369 strenthening constantly. Through molecular interaction research, the key events of this feedback loop were found, which mainly about c-Rel down-regulates miR-133a-3p by binding its precursor promoter sequence, down-regulation of miR-133a-3p can continue to inhibit the expression of mir-379 ~ 369 cluster members including miR-329-5p which targeting 3’UTR of *Rel* itself and its positive regulator *Akt3*. So far, After HPV16 infection, the network that abnormally raises the transcription factor c-Rel caused by early gene E5 and strengthening by down-regulating miR-133a-3p through an intensifying feedback loop leading to the subsequent decrease of a series tumor suppressive miRNAs of mir-379-369 cluster has been clearly mapped. With such a network, c-Rel acts on the whole process of occurrence and progression of cervical cancer during early and late HPV16 infection by affecting cell proliferation, apoptosis, and autophagy and so on. About these HPV16 related miRNAs, although there were some studies about a few of them in cervical cancer reported previously [25–27], this regulatory network and its function, regulation mechanism on cervical cancer is presented firstly. Thus, our study provided the latest interpretation of the pathogenesis and progression of cervical cancer.

## Supplementary Information

**Additional file 1:Table S1.** Primer sequences of the genes used in this study. **Table S2.** The synthetic sequencesused in this study. Table S3. The candidate transcription factors of each HPV16 related miRNA. **Figure S3.** A Normalized expression of HPV16 E5,E6 and E7 after transfected overexpression vector of E5,E6 and E7 or empty vector P2K7 in C33-A cells respectively. B:Normalized expression of HPV16 E5,E6 and E7 after transfected shRNA of E5,E6 and E7 or empty vector H1 in SiHa cells respectively. C:Normalized expression oftranscription factors *Rel,* *Nfκb1*and *Rela* after transfected overexpression vector of *Rel, Nfκb1*and *Rela* or empty vector P2K7 in C33-A cells respectively. D: Normalized expression oftranscription factors *Rel*, *Nfκb1*and *Rela *after transfected shRNAof *Rel*, *Nfκb1*and *Rela *or empty vector P2K7 H1 in SiHa cells respectively.

## Data Availability

The data that support the findings of this study are available from the corresponding author upon reasonable request.

## References

[1] Bray F, Ferlay J, Soerjomataram I, Siegel RL, Torre LA, Jemal A (2018). Global cancer statistics 2018: GLOBOCAN estimates of incidence and mortality worldwide for 36 cancers in 185 countries. CA Cancer J Clin.

[2] Vonsky M, Shabaeva M, Runov A, Lebedeva N, Chowdhury S, Palefsky JM (2019). Carcinogenesis associated with human papillomavirus infection mechanisms and potential for immunotherapy. Biochem Biokhimiia.

[3] den Boon JA, Pyeon D, Wang SS, Horswill M, Schiffman M, Sherman M (2015). Molecular transitions from papillomavirus infection to cervical precancer and cancer: role of stromal estrogen receptor signaling. Proc Natl Acad Sci USA.

[4] Konopnicki D, Manigart Y, Gilles C, Barlow P, De Marchin J, Feoli F (2016). High-risk human papillomavirus genotypes distribution in a cohort of HIV-positive women living in Europe: epidemiological implication for vaccination against human papillomavirus. AIDS.

[5] Liu C, Lin J, Li L, Zhang Y, Chen W, Cao Z (2015). HPV16 early gene E5 specifically reduces miRNA-196a in cervical cancer cells. Sci Rep.

[6] Hobert O (2008). Gene regulation by transcription factors and microRNAs. Science.

[7] Vaquerizas JM, Kummerfeld SK, Teichmann SA, Luscombe NM (2009). A census of human transcription factors: function, expression and evolution. Nat Rev Genet.

[8] Ying H, Lv J, Ying T, Li J, Yang Q, Ma Y (2013). MicroRNA and transcription factor mediated regulatory network for ovarian cancer: regulatory network of ovarian cancer. Tumour Biol.

[9] Ye H, Liu X, Lv M, Wu Y, Kuang S, Gong J (2012). MicroRNA and transcription factor co-regulatory network analysis reveals miR-19 inhibits CYLD in T-cell acute lymphoblastic leukemia. Nucleic Acids Res.

[10] Paolini F, Zaccarini M, Francesconi A, Mariani L, Muscardin L, Donati P (2020). Beta HPV Type 15 can interfere with NF-κB activity and apoptosis in human keratinocytes. Front Cellular Infect Microbiol.

[11] Chen J, Stark LA (2019). Insights into the Relationship between Nucleolar Stress and the NF-kappaB Pathway. Trends in genetics : TIG.

[12] Jahic M, Jahic E, Mulavdic M, Hadzimehmedovic A (2017). Difference between cryotherapy and follow up low grade squamous lesion of cervix uteri. Med Archives.

[13] Yu LL, Guo HQ, Lei XQ, Qin Y, Wu ZN, Kang LN (2016). p16/Ki-67 co-expression associates high risk human papillomavirus persistence and cervical histopathology: a 3-year cohort study in China. Oncotarget.

[14] Guha M, Fang JK, Monks R, Birnbaum MJ, Avadhani NG (2010). Activation of Akt is essential for the propagation of mitochondrial respiratory stress signaling and activation of the transcriptional coactivator heterogeneous ribonucleoprotein A2. Mol Biol Cell.

[15] Zhang H, Chi J, Hu J, Ji T, Luo Z, Zhou C (2021). Intracellular AGR2 transduces PGE2 stimuli to promote epithelial-mesenchymal transition and metastasis of colorectal cancer. Cancer Lett.

[16] Pakdel F, Farhadi A, Pakdel T, Andishe-Tadbir A, Alavi P, Behzad-Behbahani A (2021). The frequency of high-risk human papillomavirus types, HPV16 lineages, and their relationship with p16(INK4a) and NF-κB expression in head and neck squamous cell carcinomas in Southwestern Iran. Brazilian J Microbiol.

[17] Vandermark ER, Deluca KA, Gardner CR, Marker DF, Schreiner CN, Strickland DA (2012). Human papillomavirus type 16 E6 and E 7 proteins alter NF-kB in cultured cervical epithelial cells and inhibition of NF-kB promotes cell growth and immortalization. Virology.

[18] Xie C, Zhang LZ, Chen ZL, Zhong WJ, Fang JH, Zhu Y (2020). A hMTR4-PDIA3P1-miR-125/124-TRAF6 regulatory axis and its function in NF kappa B signaling and chemoresistance. Hepatology.

[19] Slattery ML, Mullany LE, Sakoda L, Samowitz WS, Wolff RK, Stevens JR (2018). The NF-κB signalling pathway in colorectal cancer: associations between dysregulated gene and miRNA expression. J Cancer Res Clin Oncol.

[20] El-Daly SM, Omara EA, Hussein J, Youness ER, El-Khayat Z (2019). Differential expression of miRNAs regulating NF-κB and STAT3 crosstalk during colitis-associated tumorigenesis. Mol Cell Pro.

[21] Hu YL, Feng Y, Chen YY, Liu JZ, Su Y, Li P (2020). SNHG16/miR-605-3p/TRAF6/NF-κB feedback loop regulates hepatocellular carcinoma metastasis. J Cell Mol Med.

[22] Liu DL, Lu LL, Dong LL, Liu Y, Bian XY, Lian BF (2020). miR-17–5p and miR-20a-5p suppress postoperative metastasis of hepatocellular carcinoma via blocking HGF/ERBB3-NF-κB positive feedback loop. Theranostics.

[23] Li J, Jia H, Xie L, Wang X, Wang X, He H (2009). Association of constitutive nuclear factor-kappaB activation with aggressive aspects and poor prognosis in cervical cancer. Intern J gynecol Cancer.

[24] Hua F, Li CH, Gao YC, Li J, Meng F (2019). Molecular mechanism and role of NF-κB in the early diagnosis of cervical cancer. J Balkan Union Oncol.

[25] Yuan LY, Zhou M, Lv H, Qin X, Zhou J, Mao X (2019). Involvement of NEAT1/miR-133a axis in promoting cervical cancer progression via targeting SOX4. J Cell Physiol.

[26] Zhao W, Liu Y, Zhang L, Ding L, Li Y, Zhang H (2020). MicroRNA-154–5p regulates the HPV16 E7-pRb pathway in cervical carcinogenesis by targeting CUL2. J Cancer.

[27] Xu J, Zhang J (2020). LncRNA TP73-AS1 is a novel regulator in cervical cancer via miR-329–3p/ARF1 axis. J Cell Biochem.

