# Supplementary material for: Transcription factor c-Rel regulated by E5 affects the whole process after HPV16 infection through miR-133a-modulated feedback loop aim at mir-379-369 cluster
Source: Cancer Cell Int. 2022 Dec 1;22:375. doi: 10.1186/s12935-022-02794-6 (PMC9714012; doi:10.1186/s12935-022-02794-6)
Supplement: Supplementary file 1 — Additional file 1:Table S1. Primer sequences of the genes used in this study. Table S2. The synthetic sequencesused in this study. Table S3. The candidate transcription factors of each HPV16 related miRNA. Figure S3. A Normalized expression of HPV16 E5,E6 and E7 after transfected overexpression vector of E5,E6 and E7 or empty vector P2K7 in C33-A cells respectively. B:Normalized expression of HPV16 E5,E6 and E7 after transfected shRNA of E5,E6 and E7 or empty vector H1 in SiHa cells respectively. C:Normalized expression oftranscription factors Rel, Nfκb1and Rela after transfected overexpression vector of Rel, Nfκb1and Rela or empty vector P2K7 in C33-A cells respectively. D: Normalized expression oftranscription factors Rel, Nfκb1and Rela after transfected shRNAof Rel, Nfκb1and Rela or empty vector P2K7 H1 in SiHa cells respectively. [file 12935_2022_2794_MOESM1_ESM.pdf]

Addidtional Table S1.   Primer sequences of the genes used in this study

Gene		Sequence
HPV16 E5 - qPCR	Forward	CTTGATACTGCATCCACAACA
	Reverse	AAAAGCGTGCATGTGTATGT
HPV16 E6 - qPCR	Forward	ACTTTGCTTTTCGGGATTTATGC
	Reverse	AGGACACAGTGGCTTTTGACAGTT
HPV16 E7 - qPCR	Forward	ATGCATGGAGATACACCTACATTGC
	Reverse	TTATGGTTTCTGAGAACAGATGGGG
GAPDH - qPCR	Forward	CTGTCGTGTGACCACACTGGTGCC
	Reverse	ATGCTTGTCATTTTTCATTACT
HPV16 E5 - cloning	Forward	GGTACCCACCATGACAAATCTTGATACTGCATCC
	Reverse	TCTAGATTATGTAATTAAAAAGCGTGCAT
HPV16 E6 - cloning	Forward	GGTACCCACCATGCACCAAAAGAGAACTGCAATG
	Reverse	TCTAGATTACAGCTGGGTTTCTCTCGTG
HPV16 E7 - cloning	Forward	GGTACCCACCATGCATGGAGATACACCTACATT
	Reverse	TCTAGATTATGGTTTCTGAGAACAGATGGG
Rel-qPCR	Forward	ACATGGTAATTTGACGACTGCT
	Reverse	GCTTCCCAATCGTTCAACACA
Rela-qPCR	Forward	ATGTGGAGATCATTGAGCAGC
	Reverse	CCTGGTCCTGTGTAGCCATT
Nfκb1-qPCR	Forward	GAAGCACGAATGACAGAGGC
	Reverse	GCTTGGCGGATTAGCTCTTTT
Rel -cloning	Forward	ATAAGAATGCGGCCGCTAAACTATatggcctccggtgcgtataa
	Reverse	TTGGCGCGCCAAttatacttgaaaaaattcatatggaaagga
Rela -cloning	Forward	ATAAGAATGCGGCCGCTAAACTATatggacgaactgttcccctcatc
	Reverse	TTGGCGCGCCAAttaggagctgatctgactcagcaggg
Nfκb1-cloning	Forward	ATAAGAATGCGGCCGCTAAACTATatggcagaagatgatccatatttgggaagg
	Reverse	TTGGCGCGCCAActaaattttgccttctagaggtccttcctg
HPV16 E5 - shRNA	top	GATACTGCATCCACAACATTATTCAAGAGATAATGTTGTGGATGCAGTATCTTTTTTGT
	bottom	CTAGACAAAAAAGATACTGCATCCACAACATTATCTCTTGAATAATGTTGTGGATGCAGTATC
HPV16 E6 - shRNA	top	GGAGCGACCCAGAAAGTTACCTTCAAGAGAGGTAACTTTCTGGGTCGCTCCTTTTTTGT
	bottom	CTAGACAAAAAAGGAGCGACCCAGAAAGTTACCTCTCTTGAAGGTAACTTTCTGGGTCGCTCC
HPV16 E7 - shRNA	top	GCATGGAGATACACCTACATTTTCAAGAGAAATGTAGGTGTATCTCCATGCTTTTTTGT
	bottom	CTAGACAAAAAAGCATGGAGATACACCTACATTTCTCTTGAAAATGTAGGTGTATCTCCATGC
Rel - shRNA	top	GAGCACAGCACAGACAACAACCGAATTCAAGAGATTCGGTTGTTGTCTGTGCTGTGCTCTTTTTTGT
	bottom	CTAGACAAAAAAGAGCACAGCACAGACAACAACCGAATCTCTTGAATTCGGTTGTTGTCTGTGCTGTGCTC
Rela - shRNA	top	CAGATACAGACGATCGTCACCGGATTTCAAGAGAATCCGGTGACGATCGTCTGTATCTGTTTTTTGT
	bottom	CTAGACAAAAAACAGATACAGACGATCGTCACCGGATTCTCTTGAAATCCGGTGACGATCGTCTGTATCTG
Nfκb1- shRNA	top	GGGAAACCATATGAGCCAGAGTTTATTCAAGAGATAAACTCTGGCTCATATGGTTTCCCTTTTTTGT
	bottom	CTAGACAAAAAAGGGAAACCATATGAGCCAGAGTTTATCTCTTGAATAAACTCTGGCTCATATGGTTTCCC
miR-133a1	Forward	CCTCGAGGggatttgggctgagcgggtcttgttca
	Reverse	CGACGCGTCGctctgggtcctcctgcccctctcttgact

miR-133a2	Forward	CCTCGAGGacctgtttaattgtttggttcataaagtgcac
	Reverse	CGACGCGTCGccatcagcccacactctgcaaa
miR-133a3	Forward	CCTCGAGGggccagggaggggtggactctgggagccttga
	Reverse	CGACGCGTCGagatccgaggaacagcgacgtcggtgaagaa
miR-133a1 mt	Forward	aacaagccgttttggggtgACTggGGGcctcttgccttttaattagggg
	Reverse	cccctaattaaaaggcaagaggCCCccAGTcaccccaaaacggcttgttt
miR-133a2 mt	Forward	aggggcaggaggacccagagGTTgtGGGccggagaatccacatcccaggc
	Reverse	gcctgggatgtggattctccggCCCacAACctctgggtcctcctgcccct
miR-133a3 mt	Forward	gccgcagtggcctccccgtgTTTgtGGGgcagcctgccccagtgctctgc
	Reverse	gcagagcactggggcaggctgcCCCacAAAcacggggaggccactgcggc

Additional Table S2. The synthetic sequences used in this study

Akt3 3'UTR wild binding site sequence	CCGCTCGAGCGGagctgactgttcttgcaggggccacttgcttctcctagagtacaaaagta agggccttccttactaactgcagggtctctctattacacctcaacatacacactttgctgctactgtt tgtactgtctacagtagaatttccttatcttgctcctggtagtgcattacaggcaagcatgaaatgt aaagtatttatttaaataaaaagaaaacctctaaattggtaattgaattacctccctgtagctttat agtttgtgacatttcttgaccttgctagttctttcattagatctgcgcaagatctagtcatctggttaa ggattttaagcagatgcaactataaacccaagaaactgtattactattactgttggtcatactaaa cctgtctatttcctgaagtatatgacATTTGCGGCCGCTTTA
Akt3 3'UTR mutant binding sequence	CCGCTCGAGCGGagctgactgttcttgcaggggccacttgcttctcctagagtacaaaagta agggccttccttactaactgcagggtctctctattacacctcaacatacacactttgctgctactgtt tgtactgtctacagtagaatttccttatcttgctcctggtagtgcattacaggcaagcatgaaatgt aaagtatttatttaaataaaaagccccaagctaaattggtaattgaattacctccctgtagctttat agtttgtgacatttcttgaccttgctagttctttcattagatctgcgcaagatctagtcatctggttaa ggattttaagcagatgcaactataaacccaagaaactgtattactattactgttggtcatactaaa cctgtctatttcctgaagtatatgacATTTGCGGCCGCTTTA
Rel 3'UTR wild binding site sequence	CCGCTCGAGCGGaatgtattcttaaatttcaagcaaatttaagataaaacttgtaatggctatg ccattgaaaaacttaatttttatttttgaggcccatgggccaaggtaacccctaaggggttttctta ggcttcttggagcttagatttgtatgtatatcaaaatgtctttaaaatgttaagttgggcagaaggca gttgaagtgagctttcaaggtatgggaggttttctacattttatactatttcaatctatgcctttaaagt tgcttatgattttagctgtacactcatttttaaggggaagaagtttccttggaccattcgcctttctta gatgtcctcactccctgtgatctcataaaactgcctatttgacatctctatctagaaatctaattaaag ctcacactcagcatatccaaaactgATTTGCGGCCGCTTTA
Rel 3'UTR mutant binding site sequence	CCGCTCGAGCGGaatgtattcttaaatttcaagcaaatttaagataaaacttgtaatggctatg ccattgaaaaacttaatttttatttttgaggcccatgggccaaggtaacccctaaggggttttctta ggcttcttggagcttagatttgtatgtatatcaaaatgtctttaaaatgttaagttgggcagaaggca gttgaagtgagctttcaaggtatgggcttggggctacattttatactatttcaatctatgcctttaaag ttgcttatgattttagctgtacactcatttttaaggggaagaagtttccttggaccattcgcctttctta gatgtcctcactccctgtgatctcataaaactgcctatttgacatctctatctagaaatctaattaaag ctcacactcagcatatccaaaactgATTTGCGGCCGCTTTA

Additional Table S3. The candidate transcription factors of each HPV16 related miRNA

miRNA	Transcription Factors
miR-133a-3p/5p	c-Rel、NF-KB p50、NF-KB p65
miR-133b	c-Rel、HLF
miR-196a-5p	c-Rel、HNF4A、NF-KB p65
miR-154-5p	c-Rel、STAT1、CTCF
miR-299-5p	c-Rel、STAT1、HLF、ELK1
miR-329-3p/5p	c-Rel、NF-KB p50、STAT1、HLF
miR-369-5p	c-Rel、NF-KB p50、NF-KB p65、STAT1、CTCF、YY1
miR-376b-3p	c-Rel、NF-KB p50、NF-KB p65
miR-379-5p	c-Rel、STAT1、YY1、ELK1
miR-382-5p	c-Rel、NF-KB p50、ELK1
miR-495-3p	c-Rel、NF-KB p50、NF-KB p65、STAT1、ELK1、NR2F1

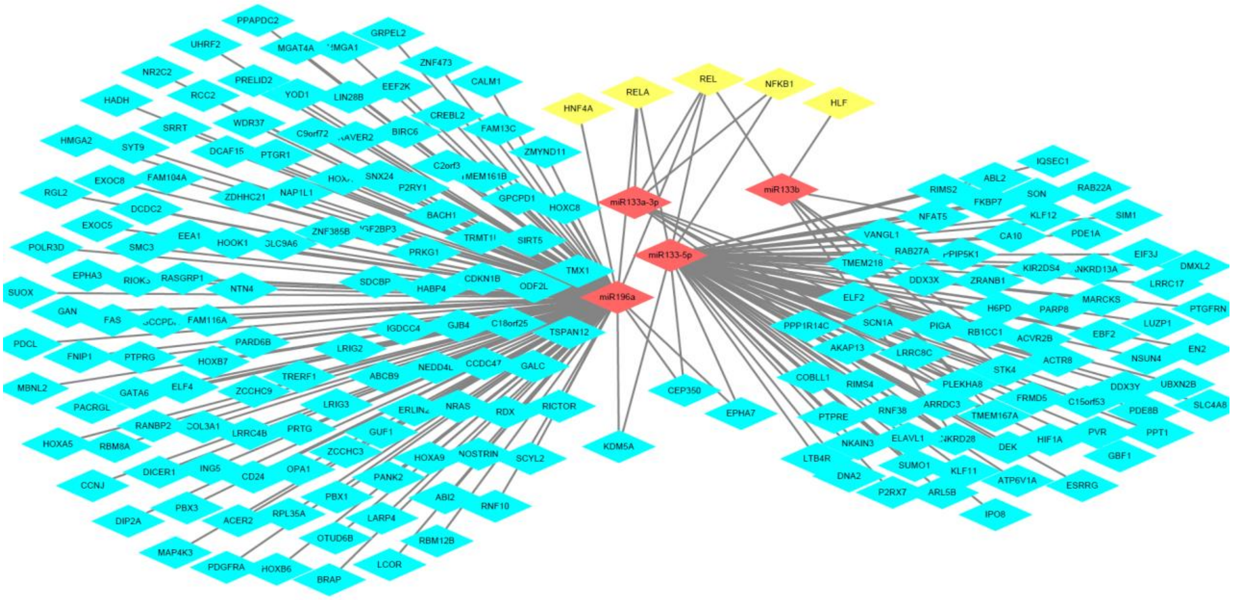

Additional Figure S1

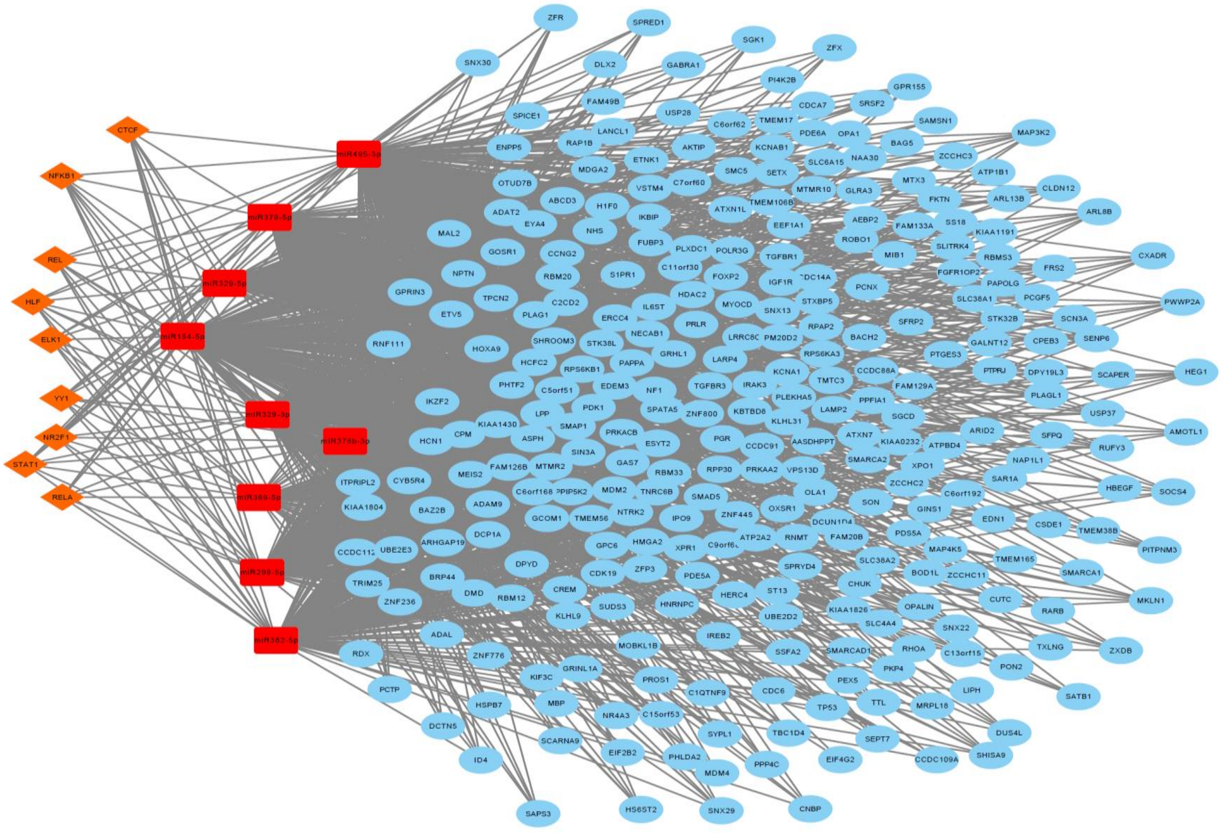

Additional Figure S2

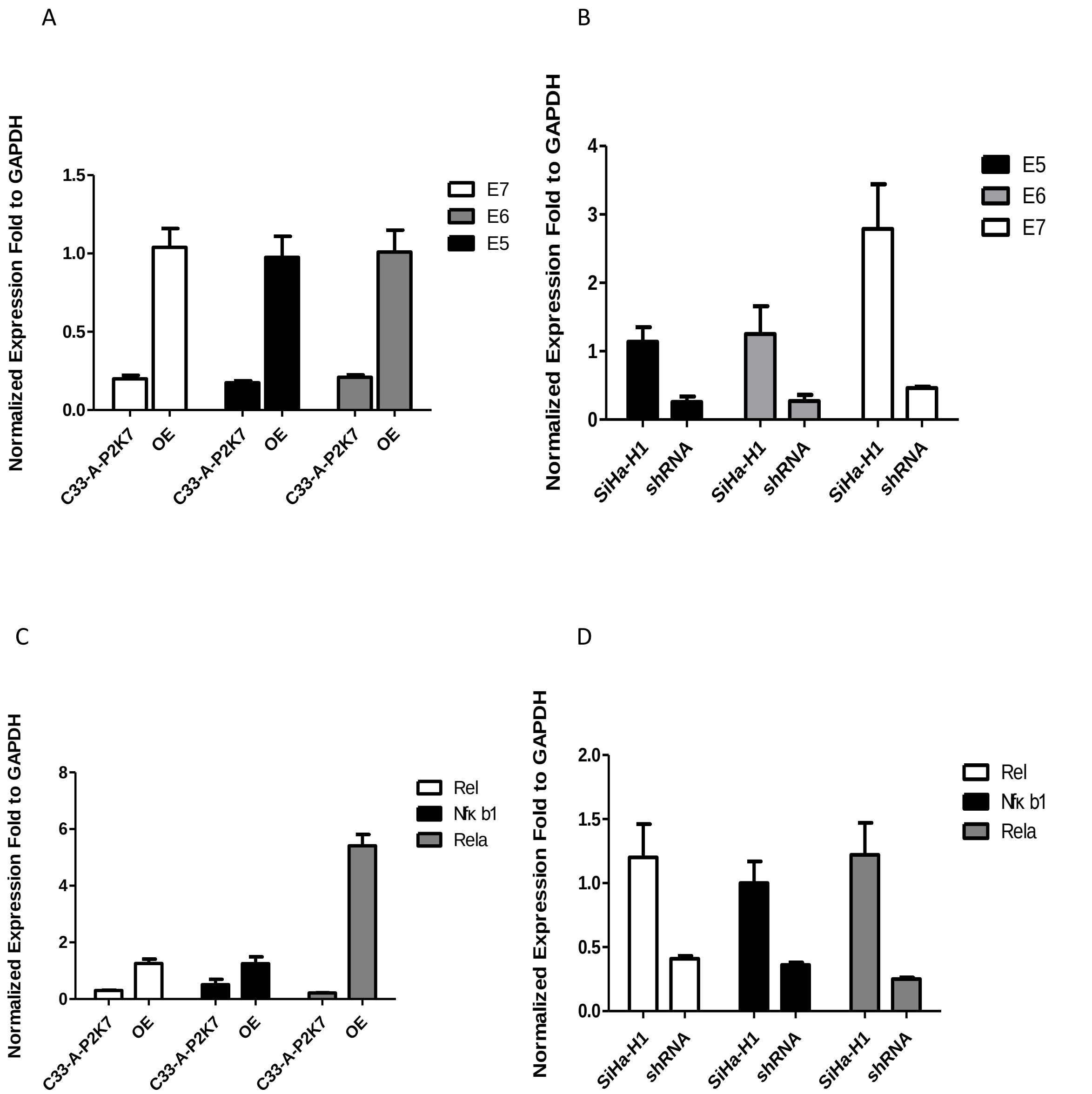

Additional Figure S3: A: Normalized expression of HPV16 E5,E6 and E7 after transfected overexpression vector of E5,E6 and E7 or empty vector P2K7 in C33-A cells respectively. B: Normalized expression of HPV16 E5,E6 and E7 after transfected shRNA of E5,E6 and E7 or empty vector H1 in SiHa cells respectively. C: Normalized expression of transcription factors *Rel*, *Nfκb1* and *Rela* after transfected overexpression vector of *Rel*, *Nfκb1* and *Rela* or empty vector P2K7 in C33-A cells respectively. D: Normalized expression of transcription factors *Rel*, *Nfκb1* and *Rela* after transfected shRNA of c- *Rel*, *Nfκb1* and *Rela* or empty vector P2K7 H1 in SiHa cells respectively.

*： $P<0.05$。
